# Insights into hospitalization pattern for drug, medicament, and biological substance poisoning, adverse effect, and underdosing in Australia: An ecological study between 1998 and 2019

**DOI:** 10.1371/journal.pone.0309362

**Published:** 2024-08-29

**Authors:** Abdallah Y. Naser

**Affiliations:** Department of Applied Pharmaceutical Sciences and Clinical Pharmacy, Faculty of Pharmacy, Isra University, Amman, Jordan; Kore University of Enna: Universita degli Studi di Enna ’Kore’, ITALY

## Abstract

**Background:**

Drug, medicament, and biological substance poisoning, adverse effects, and underdosing are significant public health concerns. Gaining insight into the patterns and trends in hospitalizations caused by these occurrences is essential for the development of preventative initiatives, optimization of treatment regimens, and improvement of patient safety. The aim of this study is to examine the trend of hospitalisation related to poisoning by, adverse effect of and underdosing of drugs, medicaments and biological substances in Australia between 1998 and 2019.

**Methods:**

This is an ecological descriptive study that examined hospitalisation related to poisoning by, adverse effect of and underdosing of drugs, medicaments and biological substances in Australia between 1998 and 2019. A nationwide hospital admissions database was used for this study.

**Results:**

Between 1998 and 2019, a total of 683,869 hospital admission episodes were recorded in Australia. The overall annual number of hospital admissions for various reasons increased by 20.5% from 29,854 in 1998 to 35,960 in 2019, representing a decrease in hospital admission rate of 10.6% [from 158.69 (95% CI 156.90–160.49) in 1998 to 141.91 (95% CI 140.44–143.37) in 2019 per 100,000 persons, trend test, p<0.05]. Overnight-stay admissions accounted for 69.2% of the total number of hospital admissions, and 30.8% were same-day admissions. Rates of same-day hospital admission decreased by 13.3% [from 50.55 (95%CI 49.54–51.57) in 1998 to 43.81 (95%CI 43.00–44.63) in 2019 per 100,000 persons]. Rates of overnight-stay hospital admission decreased by 11.1% [from 108.14 (95%CI 106.66–109.63) in 1998 to 96.17 (95%CI 94.96–97.38) in 2019 per 100,000 persons]. Admissions related to antiepileptic, sedative-hypnotic and antiparkinsonism drugs was the most prevalent hospital admissions type accounting for 26.8%. Females were responsible for 418,751 hospital admission episodes, representing 61.5% of the total number of hospital admission.

**Conclusion:**

This study found that while the overall annual number of admissions increased, the rate of admission decreased over the same period. The most common reasons for admissions were antiepileptic, sedative-hypnotic, and anti-parkinsonism drugs. The study also noted increases in admissions related to anaesthetics, therapeutic gases, hormones, and their synthetic substitutes. These findings suggest a concerning rise in the suboptimal use of these medications. In order to combat the increasing incidence of this type of admissions, it is imperative to strengthen public awareness initiatives on medicine safety and abuse.

## Introduction

Drug, medicament, and biological substance poisoning, adverse effects, and underdosing are significant public health concerns. Gaining insight into the patterns and trends in hospitalizations caused by these occurrences is essential for the development of preventative initiatives, optimization of treatment regimens, and improvement of patient safety [1, 2]. In contrast to chronic incidents, the preponderance of poisonings is categorised as acute [3–5]. Acute poisoning is a significant health issue, and acute poisoning patients are admitted to emergency departments at the hospital frequently [5]. Drug poisoning may occur either accidentally or intentionally [6]. A considerable contributor to overall healthcare costs, mortality, and morbidity is intentional and unintentional (accidental) poisoning or overdose of drugs [7].

A previous study in the United Kingdom (UK) examined all causes of hospitalization and found that 6.5% were related to injury, poisoning and certain other consequences of external causes [8]. Another study in the UK found that the annual rate of hospital admissions due to medications administration errors increased by 32.0% between 1999 and 2020 [9]. Besides, the most common three indications of hospital admissions were non-opioid analgesics, antipyretics and antirheumatics, psychotropic drugs, and antiepileptic, sedative-hypnotic and antiparkinsonism drugs [9, 10]. Another study in the UK examined paediatric hospitalisation related to medications administration errors of non-opioid analgesics, antipyretics and antirheumatics and found that the hospital admission rate increased by 12.3% for patients aged 15 years and below between 1999 and 2020 [11].

In Australia, accidental poisoning, which predominantly includes pharmaceutical drugs, results in 1,000 hospital admissions and over 1,000 mortalities annually. In Australia, accidental poisoning caused 1,500 mortality and 10,800 hospital admissions (42 per 100,000 individuals) in 2019/2020. Moreover, 83% of hospital admissions for accidental poisoning were caused by adverse pharmaceutical drug exposures in 2019/2020. Male and female accidental poisoning hospital admission rates were comparable, while male mortality rates for unintentional poisoning were over twice as high. Additionally, hospital admissions and deaths’ age distributions differed noticeably [12].

In Australia, intentional self-poisoning with anti-epileptic, sedative-hypnotic, antiparkinsonism and psychotropic medicines (including benzodiazepines) was the most frequent form of self-harm mode that required hospital admission between 2008/2009 and 2020/2021, accounting for 40% of such hospital admissions in 2020/2021. Moreover, 22% of intentional self-harm hospitalisations in 2020/2021 were due to poisoning with nonopioid analgesics, antipyretics and antirheumatics, becoming the second most prevalent form of self-harm contributing to hospital admission. As a result of intentional self-poisoning with anti-epileptic, sedative-hypnotic, antiparkinsonism, and psychiatric medications in 2020/2021, almost 8,000 females in comparison to 3,800 males were hospitalised. Additionally, compared to male hospitalisations, intentional self-poisoning with nonopioid analgesics, antipyretics, and antirheumatics hospitalisations were higher among females by over 3-fold (about 1,200 males Vs exceeding 5,300 females) in 2020/2021 [13].

Epidemiologic studies, national surveillance programs, and assessment of possible interventions are essential to address this issue [5, 14, 15]. During the past years multiple studies have been conducted to examine the hospitalisation pattern across different diseases area [8, 16–22]. However, there is no previous comprehensive study that examined hospitalisation related to poisoning by drugs, medicaments and biological substances in Australia. Thus, this study aims to examine the trend of hospitalisation related poisoning by, adverse effect of and underdosing of drugs, medicaments and biological substances in Australia.

## Materials and methods

### Study design

Between 1998 and 2019, this ecological descriptive study investigated hospitalizations in Australia attributable to drug, pharmaceutical, and biological substance toxicity, adverse effects, and underdosing. A database of hospital admissions from across the nation was utilized for this research.

### Data sources

#### National hospital morbidity database

The National Hospital Morbidity Database (NHMD) is a component of the National Hospitals Data Collection. The National Hospitals Data Collection comprises The Australian Institute of Health and Welfare’s (AIHW) preeminent national hospital datasets [13]. NHMD is an internet-based repository that compiles data supplied by health authorities in Australian states and territories [23]. Episode-level records obtained from morbidity data collection systems of patients admitted to private and public institutions in Australia comprise the data gathered at the NHMD. The data provided is derived from the National Minimum Data Set (NMDS) for Admitted Patient Care. It includes the following information: patient diagnoses, external causes of poisoning and injury, length of hospital stay and administrative activities, procedures performed within the hospital, demographic characteristics, and length of stay. NHMD categorizes age groups as follows: less than 1 year, 1–4 years, 5–9 years, 10–14 years, 15–19 years, 20–24 years, 25–29 years, 30–34 years, 35–39 years, 40–44 years, 45–49 years, 50–54 years, 55–59 years, 60–64 years, 65–69 years, 70–74 years, 75–79 years, 80–84 years, and 85 years and over.

The objective of the NMDS for admitted patient care is to amass information regarding the conditions under which hospitalized patients in Australian institutions are treated. Episodes of care for hospital admission patients in all alcohol and substance treatment centers, freestanding day hospital facilities, private and public psychiatric and acute hospitals are included in the coverage of the NMDS. While not all hospitals, including those administered by the Australian Defence Force, corrections authorities, or Australia’s offshore territories, are encompassed within the scope of NMDS. The term "bed-day" or "inpatient day" refers to a period of time when an individual is hospitalized as an inpatient and is restricted to a bed, spending the night in the facility. The term "overnight-stay admitted care" refers to the provision of medical attention to a patient who is admitted to the hospital for a minimum of one night and is discharged on separate dates.

#### Australian Bureau of Statistics

The Australian Bureau of Statistics (ABS), which serves as the official repository of dependable and impartial data, is the Australian National Statistics Agency [24]. The ABS was utilized to gather mid-year population data spanning from 1998 to 2019. The historical population was utilized from 1998 to 2016 to obtain population data [25]. Population data were gathered via national, state, and territory populations from 2017 to 2019 [26].

#### Study population

The study incorporated all admissions data pertaining to poisoning, adverse effects, and underdosing of pharmaceuticals, medicaments, and biological substances in Australia, both in private and public settings, spanning the years 1998 to 2019. This consists of all NHMD data available in principal diagnosis data packages [27]. For the purpose of categorizing hospital admission episodes involving poisoning, adverse effects, and underdosing of medications, medicaments, and biological substances, we employed the Tenth Revision of the International Statistical Classification of Diseases and Related Health Problems (ICD-10) (T36-T50).

### Ethics statement

This study was approved by the scientific research ethic committee at Isra University, Amman, Jordan (SREC/24/03/096). Informed consent was waived by the ethics committee.

### Statistical analysis

For all analyses, SPSS version 29 (IBM Corp., Armonk, NY, USA) was utilized. In order to calculate hospitalization rates with 95% CIs, hospitalization episodes were divided by the midyear population. The study employed the Pearson chi-square test for independence to examine the variability in hospitalization rates spanning the years 1998 to 2019.

## Results

### The overall trends in hospital admissions

Between 1998 and 2019, a total of 683,869 hospital admission episodes were recorded in Australia. The overall annual number of hospital admissions for various reasons increased by 20.5% from 29,854 in 1998 to 35,960 in 2019, representing a decrease in hospital admission rate of 10.6% [from 158.69 (95% CI 156.90–160.49) in 1998 to 141.91 (95% CI 140.44–143.37) in 2019 per 100,000 persons, trend test, p<0.05].

**Fig 1:**
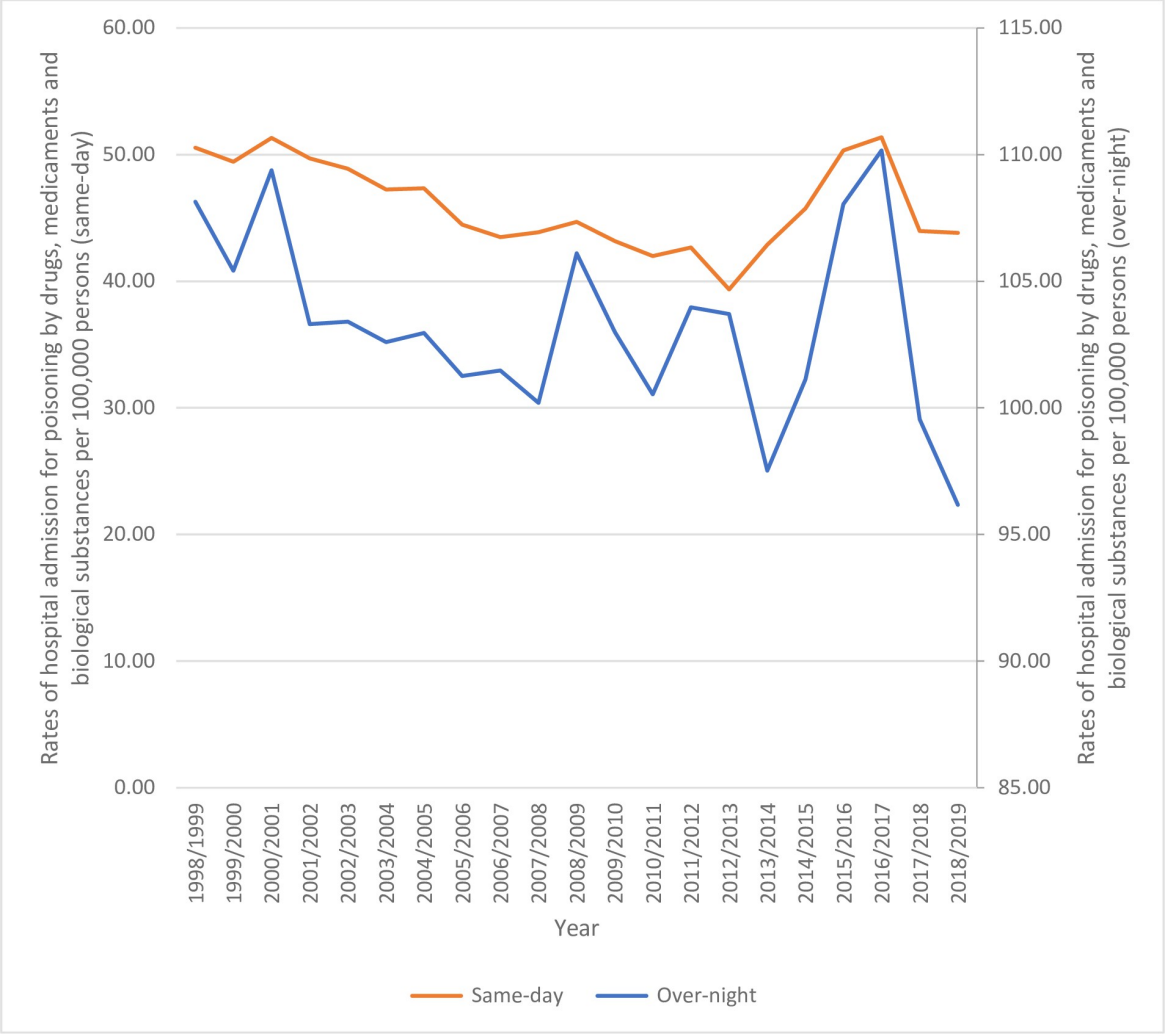
Same-day and overnight-stay hospital admission rates.

Overnight-stay episodes accounted for 69.2% of the total number of hospital admissions, and 30.8% were same-day episodes. Rates of same-day hospital admission decreased by 13.3% [from 50.55 (95%CI 49.54–51.57) in 1998 to 43.81 (95%CI 43.00–44.63) in 2019 per 100,000 persons]. Rates of overnight-stay hospital admission decreased by 11.1% [from 108.14 (95%CI 106.66–109.63) in 1998 to 96.17 (95%CI 94.96–97.38) in 2019 per 100,000 persons] (Fig 1).

Admissions related to poisoning by, adverse effect of and underdosing of antiepileptic, sedative-hypnotic and antiparkinsonism drugs was the most prevalent hospital admissions type accounting for 26.8% of the total number of hospital admissions, followed by psychotropic drugs, not elsewhere classified with 25.9%, nonopioid analgesics, antipyretics and antirheumatics with 20.4%, and narcotics and psychodysleptics [hallucinogens] with 10.5% (Table 1).

**Table 1:** Percentage of hospital admission from total number of admissions.

International Statistical Classification of Diseases system code	Description of admission cause	Percentage from total number
T36	“Systemic antibiotics”	0.5%
T37	“Other systemic anti-infectives and antiparasitics”	0.3%
T38	“Hormones and their synthetic substitutes and antagonists”	2.5%
T39	“Nonopioid analgesics, antipyretics and antirheumatics”	20.4%
T40	“Narcotics and psychodysleptics [hallucinogens]”	10.5%
T41	“Anaesthetics and therapeutic gases”	0.8%
T42	“Antiepileptic, sedative-hypnotic and antiparkinsonism drugs”	26.8%
T43	“Psychotropic drugs”	25.9%
T44	“Drugs primarily affecting the autonomic nervous system”	2.0%
T45	“Primarily systemic and haematological agents, not elsewhere classified”	2.5%
T46	“Agents primarily affecting the cardiovascular system”	3.0%
T47	“Agents primarily affecting the gastrointestinal system”	0.4%
T48	“Agents primarily acting on smooth and skeletal muscles and the respiratory system”	0.5%
T49	“Topical agents primarily affecting skin and mucous membrane and by ophthalmological, otorhinolaryngological and dental drugs”	0.8%
T50	“Diuretics and other and unspecified drugs, medicaments and biological substances”	3.0%

### Trends in hospital admissions stratified by cause

**Fig 2:**
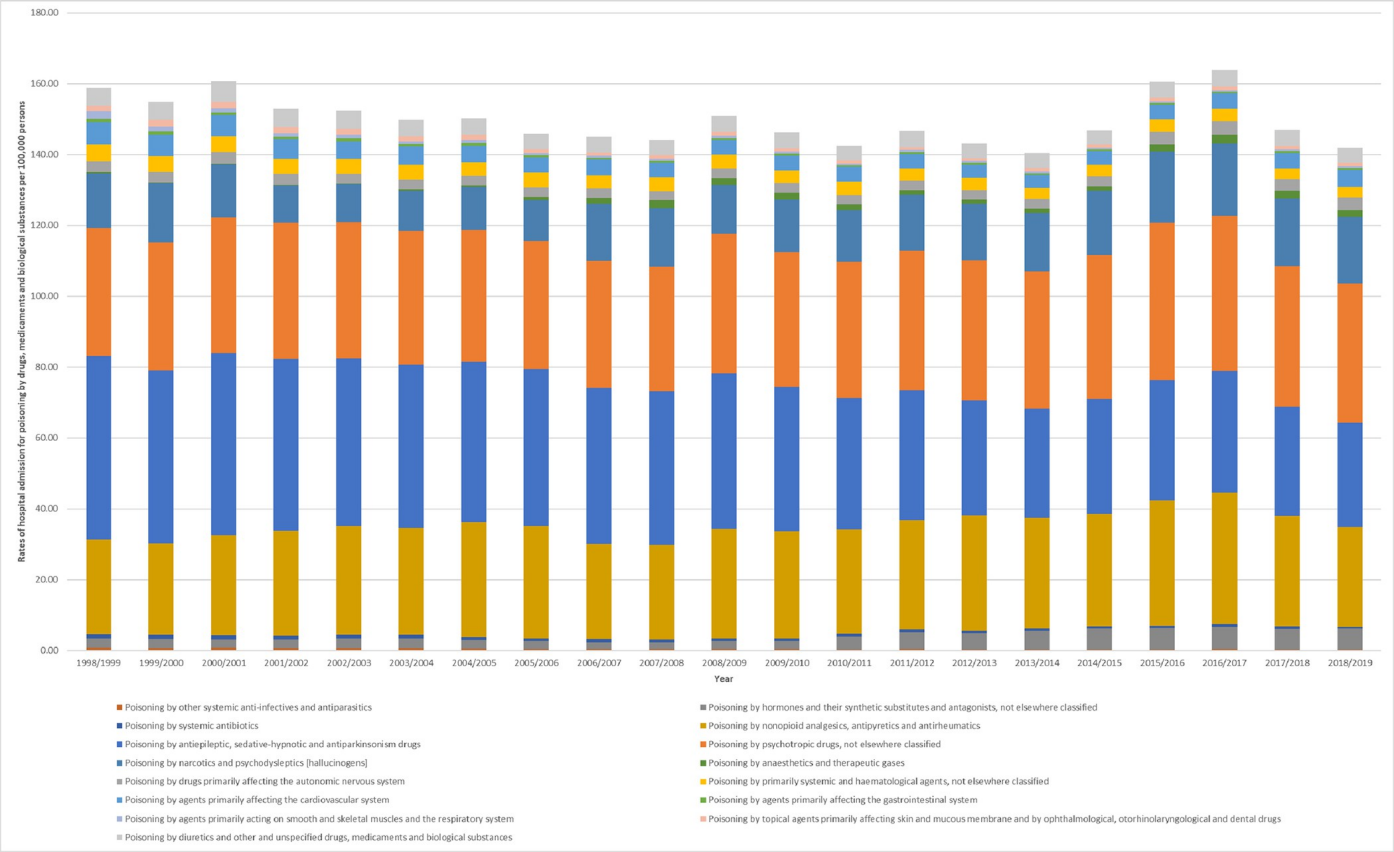
Rates of hospital admission in Australia stratified by type.

During the study time, a tremendous increase in hospital admissions rate was observed in admissions related to anaesthetics and therapeutic gases and hormones and their synthetic substitutes and antagonists, not elsewhere classified by 8.05-fold and 1.32-fold, respectively. Furthermore, hospital admissions rate for narcotics and psychodysleptics [hallucinogens drugs primarily affecting the autonomic nervous system, psychotropic drugs, and nonopioid analgesics, antipyretics and antirheumatics increased by 21.7%, 18.7%, 8.5%, and 5.7%, respectively. However, hospital admissions rate for agents primarily acting on smooth and skeletal muscles and the respiratory system, other systemic anti-infectives and antiparasitics, systemic antibiotics, antiepileptic, sedative-hypnotic and antiparkinsonism drugs, agents primarily affecting the gastrointestinal system, primarily systemic and haematological agents, topical agents primarily affecting skin and mucous membrane, and ophthalmological, otorhinolaryngological and dental drugs, agents primarily affecting the cardiovascular system, and diuretics and other and unspecified drugs, medicaments and biological substances decreased by 75.4%, 69.1%, 61.7%, 43.3%, 38.3%, 38.1%, 36.3%, 23.2%, and 16.4%, respectively (Table 2, Fig 2).

**Table 2:** Percentage change in the hospital admission rates from 1998–2019 in Australia.

Description of admission cause	Rate of admissions in 1998 per 100,000 persons (95% CI)	Rate of admissions in 2019 per 100,000 persons (95% CI)	Percentage change from 1998–2019
“Systemic antibiotics”	1.31 (1.14–1.47)	0.50 (0.41–0.59)	-61.7%
“Other systemic anti-infectives and antiparasitics”	0.77 (0.64–0.89)	0.24 (0.18–0.30)	-69.1%
“Hormones and their synthetic substitutes and antagonists”	2.55 (2.32–2.78)	5.92 (5.62–6.22)	132.2%
“Nonopioid analgesics, antipyretics and antirheumatics”	26.72 (25.98–27.46)	28.24 (27.59–28.90)	5.7%
“Narcotics and psychodysleptics [hallucinogens]”	15.51 (14.94–16.07)	18.86 (18.33–19.40)	21.7%
“Anaesthetics and therapeutic gases”	0.20 (0.14–0.27)	1.83 (1.66–1.99)	804.5%
“Antiepileptic, sedative-hypnotic and antiparkinsonism drugs”	51.68 (50.66–52.71)	29.32 (28.65–29.99)	-43.3%
“Psychotropic drugs”	36.22 (35.36–37.08)	39.29 (38.52–40.06)	8.5%
“Drugs primarily affecting the autonomic nervous system”	3.04 (2.79–3.28)	3.60 (3.37–3.84)	18.7%
“Primarily systemic and haematological agents”	4.84 (4.53–5.16)	3.00 (2.78–3.21)	-38.1%
“Agents primarily affecting the cardiovascular system”	6.26 (5.90–6.62)	4.81 (4.54–5.08)	-23.2%
“Agents primarily affecting the gastrointestinal system”	0.98 (0.84–1.12)	0.60 (0.51–0.70)	-38.3%
“Agents primarily acting on smooth and skeletal muscles and the respiratory system”	2.08 (1.88–2.29)	0.51 (0.42–0.60)	-75.4%
“Topical agents primarily affecting skin and mucous membrane and by ophthalmological, otorhinolaryngological and dental drugs”	1.47 (1.29–1.64)	0.94 (0.82–1.05)	-36.3%
“Diuretics and other and unspecified drugs, medicaments and biological substances”	5.08 (4.75–5.40)	4.25 (3.99–4.50)	-16.4%

### Trends in hospital admissions (based on gender)

**Fig 3:**
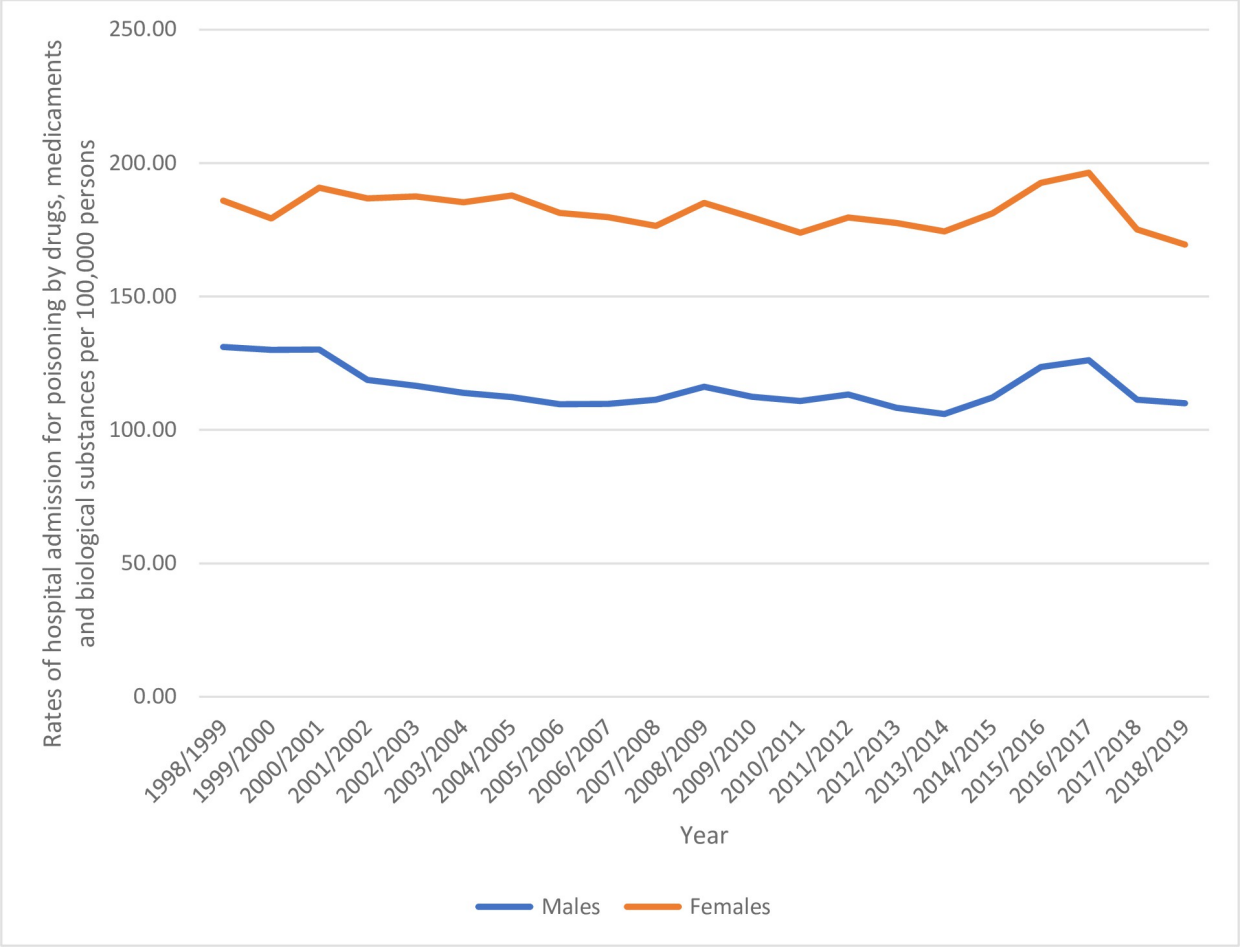
Rates of hospital admission stratified by gender.

Females were responsible for 418,751 hospital admission episodes, representing 61.5% of the total number of hospital admission, with a mean of 19,941 episodes per year. Hospital admission rate between males decreased by 16.1% [from 131.08 (95% CI 128.76–133.40) in 1998 to 109.98 (95% CI 108.15–111.81) in 2019 per 100,000 persons]. Hospital admission rate between females decreased by 8.9% [from 185.92 (95% CI 183.18–188.67) in 1998 to 169.45 (95% CI 167.19–171.70) in 2019 per 100,000 persons] (Fig 3).

### Trends in overall hospital admissions (based on age group)

**Fig 4:**
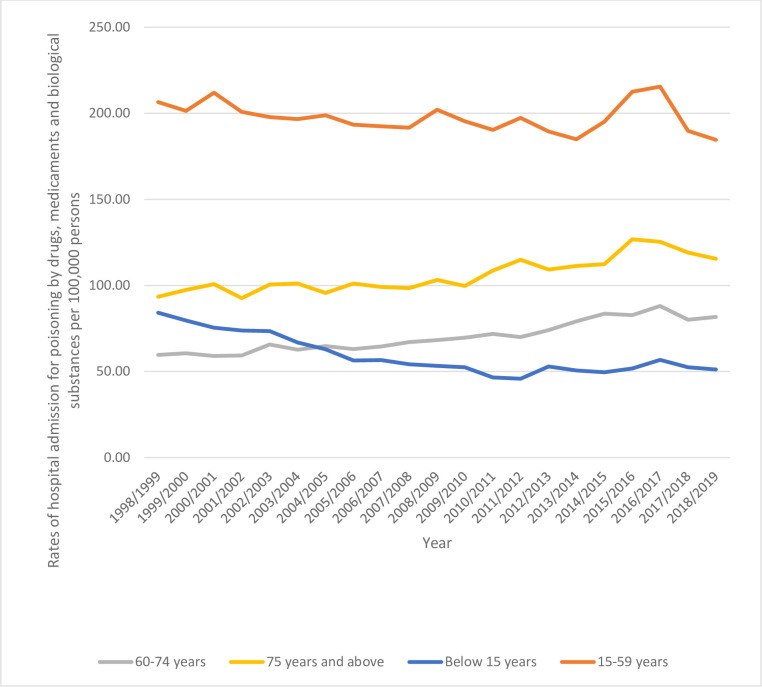
Rates of hospital admission stratified by age group.

Concerning age group diversity for hospital admission, the age group 15–59 years accounted for 81.7% of the total number of hospital admissions, followed by the age group below 15 years with 7.7%, the age group 60–74 years with 6.1%, and then the age group 75 years and above with 4.5%. Rates of hospital admission among patients aged below 15 years decreased by 39.1% [from 84.17 (95%CI 81.30–87.04) in 1998 to 51.22 (95%CI 49.18–53.26) in 2019 per 100,000 persons]. Rates of hospital admission among patients aged 15–59 years decreased by 10.6% [from 206.51 (95%CI 203.92–209.10) in 1998 to 184.58 (95%CI 182.42–186.74) in 2019 per 100,000 persons]. Rates of hospital admission among patients aged 60–74 years increased by 37.1% [from 59.62 (95%CI 56.30–62.95) in 1998 to 81.76 (95%CI 78.84–84.68) in 2019 per 100,000 persons]. Rates of hospital admission among patients aged 75 years and above increased by 23.6% [from 93.48 (95%CI 87.55–99.41) in 1998 to 115.53 (95%CI 110.48–120.57) in 2019 per 100,000 persons] (Fig 4).

### Trends in hospital admissions (based on sub-age group and gender)

**Fig 5:**
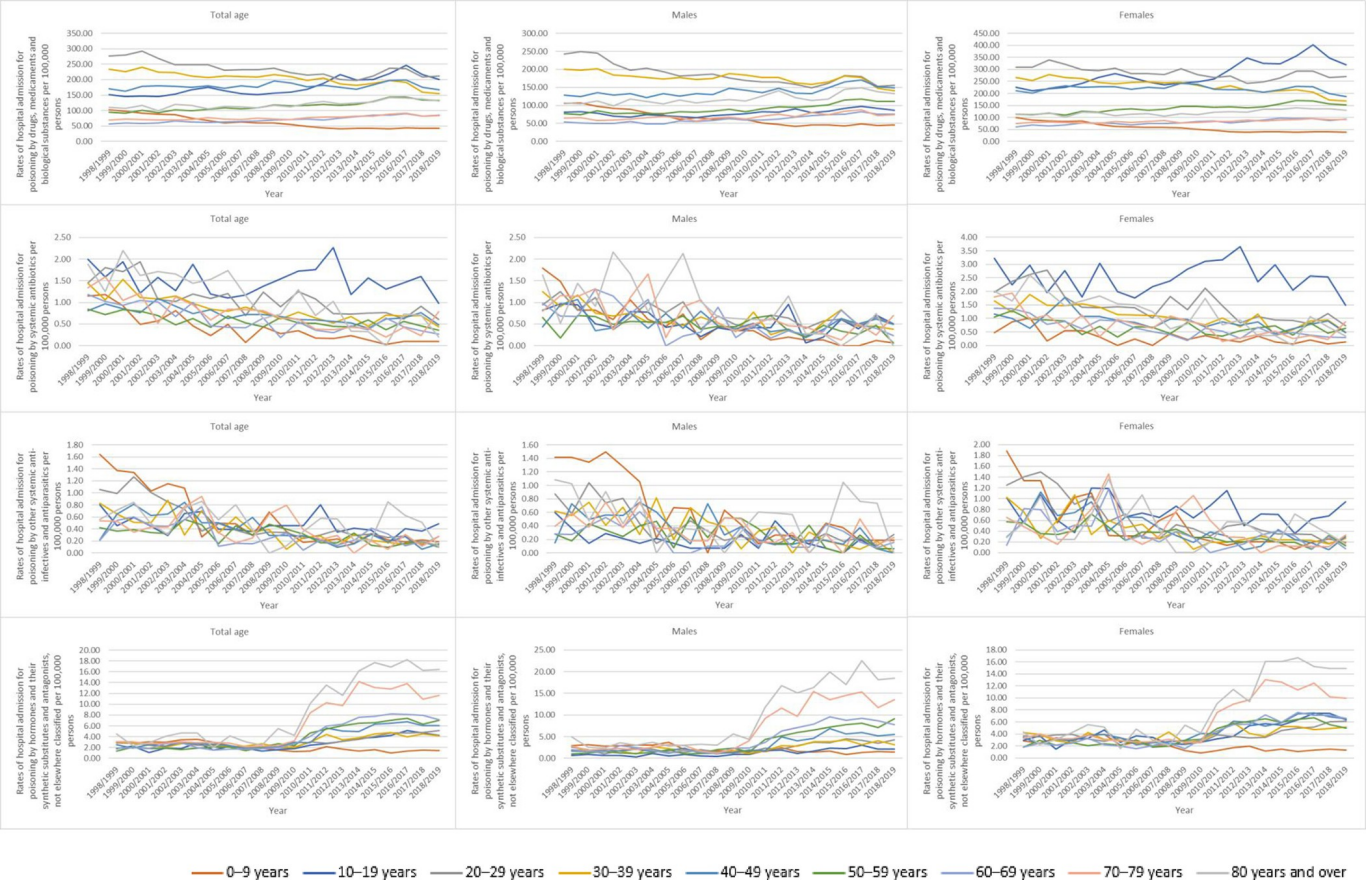
Rates of hospital admission stratified by age group and gender.

During the study time, hospital admissions rates for the total population, male, and female patients were more common among the aged 20–29 years. However, hospital admissions rates for several causes for the total population, male, and female patients were more common among the aged 80 years and over, that include: hormones and their synthetic substitutes and antagonists, drugs primarily affecting the autonomic nervous system, primarily systemic and haematological agents, agents primarily affecting the cardiovascular system, agents primarily affecting the gastrointestinal system, and diuretics and other and unspecified drugs, medicaments and biological substances (Table 3, Fig 5).

**Table 3:** Age groups that have the highest rate of admissions stratified by cause.

Description of admission cause	Age with highest rate of admissions		
	Total	Males	Females
**“Systemic antibiotics”**	10–19 years	80 years and over	10–19 years
**“Other systemic anti-infectives and antiparasitics”**	0–9 years	0–9 years	10–19 years
**“Hormones and their synthetic substitutes and antagonists”**	80 years and over		
**“Nonopioid analgesics, antipyretics and antirheumatics”**	10–19 years	20–29 years	10–19 years
**“Narcotics and psychodysleptics [hallucinogens]”**	20–29 years	30–39 years	20–29 years
**“Anaesthetics and therapeutic gases”**	20–29 years		
**“Antiepileptic, sedative-hypnotic and antiparkinsonism drugs”**	30–39 years	30–39 years	40–49 years
**“Psychotropic drugs”**	20–29 years		
**“Drugs primarily affecting the autonomic nervous system”**	80 years and over		
**“Primarily systemic and haematological agents”**	80 years and over		
**“Agents primarily affecting the cardiovascular system”**	80 years and over		
**“Agents primarily affecting the gastrointestinal system”**	80 years and over		
**“Agents primarily acting on smooth and skeletal muscles and the respiratory system”**	0–9 years		
**“Topical agents primarily affecting skin and mucous membrane and by ophthalmological, otorhinolaryngological and dental drugs”**	0–9 years		
**“Diuretics and other and unspecified drugs, medicaments and biological substances”**	80 years and over		

## Discussion

Poisoning, adverse effect, and underdosing can manifest in several forms, including acute and chronic incidents. It can be caused by multiple circumstances and have various associated consequences [3]. This study aimed to investigate the trend in hospital admissions in Australia specifically caused by poisoning by, and adverse effect of drugs, medicaments, and biological substances in Australia.

This study revealed a 20.5% increase in the total yearly hospital admissions. The figure rose from 29,854 in 1998 to 35,960 in 2019. The increase in the number of poisoning incidents is justified by the corresponding increase in the population during this period. Specifically, the Australian population rose from 18 million in 1996 to 24 million in 2015 [28]. However, despite the increase in population, this study found that the rate of hospital admissions decreased by 10.6% between 1998 and 2019. This decline is consistent with a similar study conducted in Slovenia [7]. Overnight-stay admissions accounted for 69.2% of all hospital admissions. This represents an 11.1% decrease in the rate of overnight-stay admissions. Same-day admissions accounted for 30.8%, with a decrease of 13.3% in the rate of such admissions. The management of this type of admissions, particularly within the first hour, is crucial in determining the severity of the condition, the rate of recovery, and the length of hospital stay [29, 30]. A study conducted in China found that the majority of similar types of admitted patients stay in the hospital for 48–72 hours, depending on the need for observation and doctors’ orders [31].

The primary cause of hospital admissions for poisoning by, adverse effect of and underdosing of drugs, medicaments, and biological substances is the intake of antiepileptic, sedative-hypnotic, and anti-parkinsonism drugs, which accounts for 26.8% of all such admissions. This type of admissions is particularly hazardous as it affects the respiratory system and can lead to respiratory insufficiency [32]. It can occur as a result of accidental or intentional drug abuse [33], accidental intoxication due to inadequate communication between physician and patient resulting in an overdose [34], or as a means of suicidal attempts [34]. Meanwhile, admissions caused by psychotropic drugs, which are not classified elsewhere, accounted for 25.9% of hospital admissions in Malaysia [35]. This type of admissions is primarily caused by antidepressants, overdose of tetracycline, and abuse of psychostimulant drugs [10]. Furthermore, admissions caused by nonopioid analgesics, antipyretics, and antirheumatics accounted for 20.4% of cases. This type of admissions is particularly prevalent among young children [36], and it can be attributed to the growing use of over-the-counter medications [37]. Consequently, there has been an increase in the occurrence of admissions related to these medications. In addition, poisoning caused by narcotics and psychodysleptics (hallucinogens) accounted for 10.5% of cases. These substances are a significant cause of poisoning that requires emergency department treatment. The poisoning occurs as a result of increased legal or illegal use of narcotics and psychodysleptics for pain management and other medical conditions.

During the study period, the rate of hospital admissions for poisoning by, adverse effect of and underdosing of anaesthetics and therapeutic gases increased significantly by 8.05-fold and 1.32-fold, respectively. This increase in hospital admissions for anaesthetics and therapeutic gases is influenced by various factors, including the method of administering anaesthetic drugs. Local anaesthetics, for example, can cause significant toxicity to the central nervous system and heart [38]. Additionally, while therapeutic gases have many benefits, they can be highly toxic and poisonous at higher doses [39]. The recent expansion in their use has led to an increase in the incidence of admissions related to therapeutic gases and the need for hospital admission. Hormones play a crucial role in poisoning, with various factors contributing to hormone toxicity. For example, sex hormones and sex-related hormones can be toxic to the liver and may cause hepatotoxicity, along with other associated factors [40]. Similarly, corticosteroids can be toxic in certain situations, such as long-term use leading to osteoporosis [41].

In addition, the rate of hospital admissions for poisoning by, adverse effect of and underdosing of narcotics and hallucinogens increased by 21.7% due to the increased abuse of narcotics, particularly among young individuals with unhealthy alcohol consumption habits [42, 43]. The rate of hospital admissions caused by drugs that primarily affect the autonomic nervous system increased by 18.7%. This increase is primarily due to the use of certain medications that affect the autonomic nervous system. When a large number of compounds and medications affect the functions of the autonomic nervous system, it can lead to such type of admissions. Additionally, certain drugs can cause poisoning if taken in excessive amounts, which has resulted in a higher demand for hospital admissions [44]. Furthermore, there has been an 8.5% increase in hospital admissions for poisoning by psychotropic drugs, not elsewhere classified. This increase is even higher in the UK, where the hospital admissions rate for this type accounts for 19.9%. These increases can be attributed to the growing misuse of these medications [10]. On the other hand, there has been a 5.7% increase in hospital admissions for nonopioid analgesics, antipyretics, and antirheumatics. Interestingly, this contradicts the findings of another study in Australia, where the admission rate for these medications is actually declining [36]. However, it is important to note that there is still a predominant increase in other countries [45, 46].

The hospital admissions rate for poisoning by, adverse effect of and underdosing of drugs, medicaments, and biological substances, specifically those that primarily affect smooth and skeletal muscles and the respiratory system, as well as other systemic anti-infectives and antiparasitic agents, and systemic antibiotics, has decreased by 75.4%, 69.1%, and 61.7% respectively. This decrease in hospital admissions rate can be attributed to the increased focus on addressing the causes and effects of poisonings, whether due to drug overdose or misuse [47].

Females accounted for 61.5% of hospital admissions. In Slovenia, intentional poisoning cases involving drugs, medicaments, and biological substances were predominantly reported among females [7]. Several factors, such as exposure to multiple medications, non-adherence to medication, and self-harm attempts, contribute significantly to the difference in toxicity and poisoning rates between males and females [48]. Additionally, the hospital admission rate for among females decreased by 8.9%, while the rate among males decreased by 16.1%. Furthermore, males were more commonly affected by poisoning as teenagers, whereas females constituted the majority of reported poisonings according to the American Association of Poison Control Centers [49].

In terms of age group diversity for hospital admissions, the 15–59 year age group accounted for 81.7% of the total number of admissions. The age group below 15 years accounted for 7.7% of admissions, with children below three years and below six years representing a higher number of admissions. This information is based on data from the American Association of Poison Control Centers [49]. Furthermore, the study found that hospital admissions rates for poisoning by, adverse effect of and underdosing of drugs, medicaments, and biological substances were higher among individuals aged 20–29 years. This trend was observed for both males and females, and is consistent with a similar study conducted in China where the 20–29 age group was the most affected by poisoning, whether accidental or intentional, among both genders [31]. Nevertheless, the hospitalization rates for various types of poisoning by, adverse effect of and underdosing of medications, medicaments, and biological substances were higher among those aged 80 years and older, regardless of gender.

Based on the findings of this study and in order to reduce the burden of medication errors and their associated hospitalization, healthcare professionals should ensure that the 5 rights of medication (right drug, right dose, right route, right time, and right person) are being followed, follow proper medication reconciliation procedures, ensure proper documentation for everything they do, enurse proper medication storage, update their information on proper medication administration guidelines, and use drug guide through their practices [50].

This study has limitations. Due to the limitations of publicly accessible web-based information, this study was unable to analyze patient-level data and study the underlying causes of the observed trends. This analysis did not take into account factors such as demographics, comorbidities, polypharmacy, and lifestyles due to the nature of publically available data; which caused difficulty in controlling confounding variables. Hospitalization data might include multiple hospitalization episodes for the same patients; which might have contributed to hospital admissions rate overestimation.

## Conclusion

This study investigated trends in hospital admissions related to poisoning by, adverse effect of and underdosing of drugs, medicaments, and biological substances in Australia from 1998 to 2019. It found that while the overall annual number of admissions increased, the rate of admission decreased over the same period. The most common reasons for admissions were antiepileptic, sedative-hypnotic, and anti-parkinsonism drugs, followed by psychotropic drugs, nonopioid analgesics, and narcotics and psychodysleptics. The study also noted increases in admissions related to anaesthetics, therapeutic gases, hormones, and their synthetic substitutes. These findings suggest a concerning rise in the abuse of these medications. In order to combat the increasing incidence of this type of admissions, it is imperative to strengthen public awareness initiatives on medicine safety and abuse. Healthcare practitioners should give high importance to educating patients about the correct use and storage of medications. Furthermore, the implementation of more stringent laws and enhanced monitoring of prescription pharmaceuticals is important in order to effectively reduce the misuse of these drugs.
